# Impact of child maltreatment on bulimic behaviors among the tunisian general population

**DOI:** 10.1192/j.eurpsy.2024.1166

**Published:** 2024-08-27

**Authors:** M. Turki, A. Hadj Ali, A. Chaaben, N. Halouani, M. A. Megdiche, S. Ellouze, J. Aloulou

**Affiliations:** Psychiatry B department, Hedi Chaker university hospital, Sfax, Tunisia

## Abstract

**Introduction:**

Child maltreatment (CM) refers to all forms of physical or psychological violence, sexual abuse, and neglect of a person under the age of 18, resulting in actual or potential harm to their health, survival, development, or dignity. It is recognized as a predictor of psychological difficulties in adulthood, such as bulimic behavior.

**Objectives:**

The aim of our study was to assess the link between CM and bulimic behaviors in the Tunisian general population.

**Methods:**

We conducted a cross-sectional, descriptive, and analytical study among Facebook group members, using an online questionnaire, from February 17, 2023, to May 26, 2023. All respondents over the age of 18 were included in the study. CM was assessed using the Childhood Trauma Questionnaire (CTQ), which provides information on five types of maltreatment: emotional abuse (EA), physical abuse (PA), sexual abuse (SA), emotional neglect (EN), and physical neglect (PN). The Bulimic Investigatory Test, Edinburgh (BITE) was used to screen and assess the intensity of bulimic behavior.

**Results:**

A total of 528 responses were included in the study. The mean age of the sample was 33.3±11.95 years. Mean AE, AP, AS, NE, NP, and overall CTQ scores were 8.30; 6.58; 6.38; 10.14; 7.26, and 49.72, respectively. A history of severe AE, AP, AS, NE, or NP was reported by 13.1%, 10.8%, 8.5%, 11.6% and 8.3% of respondents, respectively. The mean BITE score was 10.76 ±6.85 and 6.6% of our population were at high risk of developing bulimic behavior.

In the bivariate study, the BITE score was significantly correlated with all forms of MI. The strongest correlation was with AE (r=0.310; p<0.001).

In the multivariate study, only AE was associated with bulimic behaviors.

**Conclusions:**

This study highlighted a positive association between various forms of child neglect and abuse, and bulimic behaviors. It is therefore worth noting that interventions for these disorders may be more effective if they target not only the behavior itself but also underlying risk factors such as maltreatment.

**Disclosure of Interest:**

None Declared

